# Neurocognitive and neurobiological effects of low dose organophosphate exposure

**DOI:** 10.1016/j.neurot.2025.e00733

**Published:** 2025-09-22

**Authors:** Kelly M.C. Nguyen, Devyani Swami, Nandini Goyal, Mihika G. Jalwadi, Munjal M. Acharya, Janet E. Baulch

**Affiliations:** a Department of Radiation Oncology, USA; b Department of Anatomy and Neurobiology, School of Medicine, University of California, Irvine, CA, 92697-2695, USA

**Keywords:** Organophosphate, Adenosine kinase, Astrocytes, Cognitive function, Synaptic integrity

## Abstract

Organophosphates (OPs) have been used as nerve agents in the Persian Gulf Wars and terrorist attacks in Japan and the United Kingdom. While high doses of OPs may be lethal, lower dose OP exposures can lead to neurotoxic consequences for the central nervous system (CNS), which manifest as cognitive dysfunction including anxiety, depression, and learning and memory deficits. Therefore, OP toxicity poses a threat for warfighters, first responders, clean-up workers and civilians. Because the CNS effects of low dose OP exposures may be persistent, it is crucial to find countermeasures to mitigate any long-term neurological damage. This study evaluated the effect of a very low dose OP, metrifonate (MFT, 80 ​mg/kg, i.p.) and the efficacy of an orally available, small molecule adenosine kinase (ADK) inhibitor, ABT-702 (1.6 ​mg/kg, i.p., six daily doses), to ameliorate OP-induced long-term neurotoxicity. One-month post-exposure, MFT selectively impaired medial pre-frontal cortex (mPFC)-dependent executive function gauged by the puzzle box task, that was alleviated by ABT-702 treatment. MFT showed subtle effects on motor function at early (48h to one-week) post-exposure intervals without impacting spatial recognition memory or the hippocampal milieu. Dual immunofluorescence staining and volumetric quantification indicated reduced microglial activation and hypertrophic astrocytes post-ABT-702 treatment in MFT-exposed mice. ABT-702 also restored post-synaptic density protein, PSD-95, in the MFT-exposed mPFC. These data indicate that ABT-702 treatment protects mPFC-dependent function post-MFT exposure. The subtle impact of low dose MFT exposure on CNS function should be further evaluated at other time points and doses.

## Introduction

Organic esters of phosphoric acid, or organophosphates (OPs), have been widely used as fertilizers, pesticides, herbicides and fungicides. Unfortunately, OPs have also been used as nerve agents such as warfare weapons in the Persian Gulf Wars or the Tokyo subway and Matsumoto Sarin gas attacks. [1]. More recently there were also highly publicized casualties of exposures to Soviet era Novichok nerve agents in the United Kingdom [2]. As such, OP exposures represent a significant threat to military personnel and civilians [1].

OPs reversibly or irreversibly inhibit acetylcholinesterase (AChE), leading to an accumulation of the neurotransmitter acetylcholine (ACh) that can affect nicotinic or muscarinic receptor function at neuro-muscular junctions [3]. The detrimental health symptoms following exposure include muscle weakness, respiratory failure, seizures, and even death [4]. Lower dose OP exposures can lead to neurotoxic consequences for the central nervous system (CNS), which manifest as cognitive dysfunction including anxiety, depression, and learning and memory deficits [5,6]. Therefore, OP toxicity poses a significant threat for warfighters, first responders, clean-up workers and civilians alike in accident zones as well as regions of chemical warfare. Because the CNS effects of low dose or low duration OP exposures may persist for weeks or months post-exposure, or possibly never resolve, it is crucial to find countermeasures to mitigate the long-term neurological damage to improve the quality of life for survivors.

Possible countermeasures against OP poisoning include, but are not limited to, anti-muscarinic drugs, nicotinic receptor antagonist drugs, or oxime AChE reactivators [7]. While these compounds reduce or alleviate physical symptoms, there are no known alternatives to help combat the neurodegenerative pathologies that emerge subsequently. Recent findings have indicated that adenosine manipulation may represent a potential signaling pathway that can mitigate the neurological harm OP exposures can elicit [8,9]. Adenosine is a ubiquitous regulator of many important CNS processes, modulating neural activity as well as synaptic transmission [10,11]. The synaptic adenosine in the brain is primarily regulated by the astrocytic adenosine kinase (ADK), and this regulation is crucial for a multitude of behaviors [12]. In the brain, adenosine mainly acts on a set of synaptic G-protein-coupled A1 inhibitory and A2a stimulatory receptors. Prior research using rat models suggest that adenosine receptor agonists acting on A1 inhibitory receptors can provide neuroprotection against OPs by inhibiting the hyperexcitatory effects of ACh and other excitatory receptors, thereby reducing convulsions and neuropathology [12]. In the adult brain, astrocytes carefully control the levels of extracellular adenosine [13]. Astrogliosis that occurs during brain injury coincides with ADK overexpression leading to a low availability of synaptic adenosine [14]. Conversely, inhibition of ADK activity rapidly increases synaptic adenosine that modulates excitatory and inhibitory synaptic transmission to improve brain function [11,15,16]. In fact, it has been demonstrated that ADK inhibitors administered in mice post-irradiation can treat phenotypic symptoms of brain injury including neuroinflammation and cognitive dysfunction [17,18], that are similarly observed following OP exposures.

**Fig. 1:**
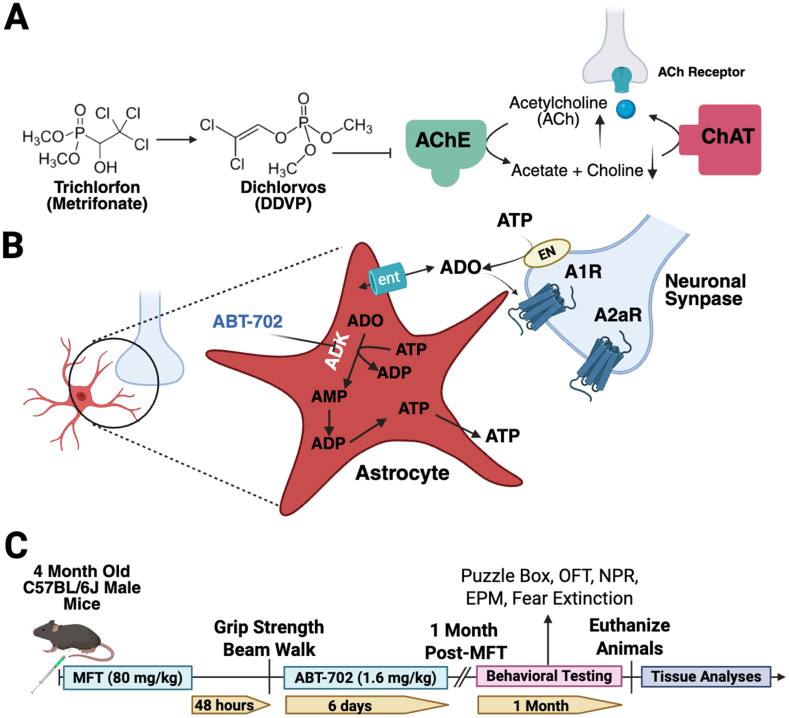
Research Design. **(A)** In the trichlorfon (metrifonate, MFT) Pathway MFT is converted to dichlorvos (DDVP) which inhibits the enzyme acetylcholinesterase (AChE) from breaking down the neurotransmitter acetylcholine (ACh) into its respective components, acetate and choline. Choline acetyltransferase (ChAT) is the enzyme responsible for synthesizing acetylcholine, which binds to corresponding ACh receptors in the central and peripheral nervous system (CNS, PNS). **(B)** Astrocytes release vesicular ATP which becomes extracellularly degraded into adenosine (ADO) by ectonucleotidases (ENs). Adenosine re-enters astrocytes through equilibrative nucleoside transporters (ENTs). When astrogliosis occurs, large quantities of adenosine are taken in and phosphorylated by cytosolic adenosine kinase (ADK), which converts ADO into downstream metabolites. ABT-702 inhibits ADK activity, thereby boosting levels of extracellular ADO that can bind to inhibitory and excitatory adenosine receptors, A1R and A2aR, respectively, on the neuronal synapses. **(C)** Experimental Timeline. Four-month-old C57Bl/6J WT male mice received a one-time injection of MFT (80 ​mg/kg, intraperitoneal), followed by a grip strength and beam walk test 48 ​h later. After the tests were performed, mice were then given a daily dose of ABT-702 (1.6 ​mg/kg, intraperitoneal) for six consecutive days. Mice were administered cognitive function tasks (Puzzle Box, PB; Open Field Test, OFT; Novel Place Recognition, NPR; elevated plus maze, EPM; and fear extinction memory consolidation) at a 1-month timepoint and then euthanized for tissue collection (graphical overview of the experiment created with BioRender.com).

Commercially used as an insecticide trichlorfon, generally known as metrifonate (MFT), is an OP compound that serves as an irreversible inhibitor of AChE. MFT acts as a prodrug in that it is not a direct inhibitor of AChE, but rather becomes nonenzymatically converted to its metabolite dichlorvos (DDVP) once taken up in the body [19] (Fig. 1A). Toxicological data reports a range of LD_50_ values (∼260 ​mg/kg – 660 ​mg/kg) for mice, dependent on sex and route of administration [20]. At the synapses, ATP is degraded into adenosine (ADO) by ectonucleotidases (ENs), which then re-enter astrocytes through equilibrative nucleoside transporters (ent; Fig. 1B). Proliferation of astrocytes, especially during periods of neuroinflammation, requires large quantities of adenosine triphosphate (ATP) and thus large quantities of adenosine to enter and become phosphorylated [21]. As an ADK inhibitor, ABT-702 (ABT) reduces levels of phosphorylated adenosine that serve as metabolites AMP, ADP and ATP, decreasing the influx of adenosine into astrocytes, leaving a greater availability of free-floating adenosine to bind to respective A1 or A2a synaptic receptors instead (A1R and A2aR, respectively) to promote neuronal survival and reduce inflammation. In the context of OP exposure, this ADK-based approach has been shown to protect against convulsion, seizure, status epilepticus, and other neuropathologies [8], [9], [22], [23]. More specifically ADK inhibition via ABT-702 or a similar compound 5-ITU has been shown to protect the mouse brain from the effects of irradiation [17], [18], modulate neural activity in rat sleep studies [24] and inhibit transient focal ischemia in rats [25]. Beyond the CNS, studies suggest ABT can be effective in reducing neuropathy and arthritis, protecting against cardiac damage and diabetes, and improving REM sleep patterns (see discussion).

In the current study, we evaluated the effect of a very low dose of MFT and the efficacy of ABT to ameliorate any observed adverse effects in the MFT-exposed brain of mice. ABT is an orally available, potent, well-tolerated small molecule that has been studied for its potential as an analgesic and anti-inflammatory agent [21,22,]. Because it can be stockpiled for emergencies, ABT is an ideal candidate to treat individuals exposed to OPs [26]. By gauging the efficacy of ABT on MFT-treated mice through behavioral testing and immunohistochemical analyses of markers of neuroinflammation, synaptic proteins and A1R and A2aR protein levels, our goal was to elucidate whether adenosine signaling is an effective target pathway to mitigate cognitive impairments post-OP exposure.

## Materials and Methods

### Animals and treatments

All animal experimentation procedures are in accordance with the guidelines provided by NIH and approved by the University of California Irvine Institutional Animal Care and Use Committee under UCI Animal Use Protocol 22–144. Wild-type male C57BL/6J mice, 15–16 weeks of age to represent the average age equivalent of a warfighter, were obtained from The Jackson Laboratory and housed in standard conditions of 20 ​°C ​± ​1 ​°C; 70 ​% ​± ​10 ​% humidity; 12h:12h light and dark cycle, in groups of 2–5 mice per cage. Experimental testing was conducted on a cohort of 56 mice (n ​= ​20 control-vehicle, n ​= ​12 ​MFT-treated, n ​= ​12 ABT-treated, and n ​= ​12 ​MFT and ABT-treated).

A 250 ​mg stock solution of MFT (BOC Sciences; Shirley, NY) was prepared in normal saline to yield a dose of 80 ​mg/kg. ABT (Tocris; Minneapolis, MN) was diluted in warm MilliQ H_2_O to yield a dose of 1.6 ​mg/kg in 2 ​% ethanol. The treatment paradigm consisted of a one-time intraperitoneal (i.p.) injection of MFT followed 48 ​h later by 6 daily i.p. injections of ABT (Fig. 1C).

### Locomotor and behavioral testing

At 48 ​h, 1 week, and 1 month post MFT exposure, mice underwent neuromuscular and locomotor function tests using the forelimb grip strength (also known as the hanging wire test) and the beam walk tests. The forelimb grip strength test was used to assess neuromuscular function for the mice [27]. Briefly, each mouse was allowed to grasp a 6 ​mm diameter beam with its front paws, and the mouse was allowed to hang freely suspended over a white tub lined to gently break its landing (40 ​cm ​× ​31.8 ​cm ​× ​15.2 ​cm). In this passive grip strength paradigm, each mouse was allowed to hang freely from the center of the beam. The latency time until the mouse dropped to the bottom of the bin was recorded by an observer blinded to the experimental groups using a timer. While no mouse achieved the goal, the maximum time a mouse would be allowed to hang was 5 ​min. Each mouse was tested 3 times, with a 1-min rest period between trials. Similarly, the beam walk assay was used to evaluate fine motor coordination and balance (as modified from Ref. [28]). For this assessment, a thicker, 8 ​mm beam was fixed across the tub and marked off into twelve 2.54 ​cm (1 in) segments. Each mouse was placed at the center of the beam and allowed to traverse freely in either direction. The number of segments (*i.e.* total distance) that the mouse traversed was manually recorded by an observer blinded to the experimental groups. For a given trial, the maximum distance a mouse traveled to be considered successful was ∼91 ​cm (36 in). As with the grip strength test each mouse was tested 3 times on the beam walk, with a 1-min rest period between trials. For both tests, the data are the mean of the 3 trials per mouse and graphically represented by those means as data points and the overall experimental cohort mean ​± ​SEM represented as columns and error bars.

Behavioral studies were initiated at ∼1-month post-MFT exposure, following the final grip strength and beam walk tests (Fig. 1C). Behavior testing occurred over ∼3 weeks and included the following paradigms in the following order: puzzle box (PB), open field testing (OFT) followed by novel place recognition (NPR). Independent investigators, blinded to the experimental groups analyzed all behavior videos. The puzzle box (PB) task is a behavioral assessment that tests a mouse's higher order executive function using tasks of increasing difficulty [29]. The testing arena is comprised of a light dark-box arena made up of two compartments divided by a plexiglass wall (25 ​× ​30 ​cm); one brightly lit start zone (40 ​cm ​× ​30 ​cm ​× ​25 ​cm) and a second smaller enclosed, dark goal zone (20 ​cm ​× ​30 ​cm ​× ​25 ​cm) with an underpass (43 ​× ​43 ​mm) between the two zones. The dark goal zone contains an igloo as incentive for the mouse to inhabit and feel protected. Mice undergo a total of 9 trials (T1 – T9) over three consecutive days, with three trials per day. The objective of each trial is that the mouse must learn to clear some obstacle blocking the opening, with the type of obstruction increasing in rigor each day.

On Day 1, trial 1 (T1) serves as a habituation period, in which there is no obstruction blocking the opening and the mouse is free to explore its environment. On T2 and T3 of Day 1, a white “u-shaped” open channel was used to narrow the underpass. On Day 2, T4 was identical to T2 and T3 in terms of the type of obstacle the mice encountered, testing the animal's recall of the previous day's task. However, for T5 and T6, the underpass was filled with 1/8″ corn cob bedding that the mouse had to dig through to reach the goal zone. On Day 3, T7 was a repetition of the previous day's T5 and T6 task, but Day 3, T8 and T9 presented the mice with a Kimwipe paper plug that had to be removed to enter the goal zone. A recall test was performed 1 month after the completion of T9, in which the mice were assessed to see if they remembered the paper plug task and reached the goal zone with a latency time similar to that observed on T9. Overall, this task sequence aimed to test native problem-solving ability upon encountering the object for the first time (T2, T5, and T8), task acquisition and reinforcement after the first attempt (T3, T6, and T9), and solution retention the following day or one month after (T4, T7, and recall). Between each trial, the boxes and igloos were cleaned with odor elimination disinfectant (Virkon, University Laboratory Animal Resources).

In the OFT, the more time the mouse spent hugging the walls of the arena, the more anxious it is thought to be, while exploring the center of the arena is a measure of exploration or boldness [30]. The open field was comprised of an acrylic box lit to ∼50–70 lux (30 ​× ​30 ​× ​30 ​cm). Mice were placed in the center of the arena and allowed to explore for 10 ​min. The total distance traveled by each mouse and the total time spent in the central zone (15 ​× ​15 ​cm) of the arena for those 10 ​min were analyzed using EthoVision XT 17 software (Noldus Information Technology, Sterling, VA). Less time spent in the central area of the box demonstrates anxiety-like behavior in the mouse. At the end of 10 ​min the animal was removed to its home cage and the arena thoroughly cleaned with ethanol.

The novel place recognition (NPR) test is a measure of hippocampal-dependent spatial memory and relies on a mouse's preference for novel stimulus as described previously [31] including testing rooms equipped with appropriate dim lighting (50–70 Lux), square arena boxes (30 ​× ​30 ​× ​30 ​cm) with a strip of blue tape down the inside of one arena wall as a cue, camera recording hardware (Noldus) and tracking software (EthoVision XT 17). For 3 consecutive days, the mice were habituated in the open field arenas with a thin layer of bedding without any objects for 10 ​min. The final day of the NPR task is a test day, comprised of two phases: a familiarization phase and a novel exploration phase. In the familiarization phase, two identical toys were magnetically secured in place 16 ​cm apart at opposing corners of the arena, and the mice were free to explore the location of these objects for 5 ​min. At the end of this phase, the mice were returned to their home cage for 5 ​min while the objects were cleansed with 10 ​% ethanol. One toy was then moved to a new, diagonal location inside the box (“novel” place), 16 ​cm from the opposite corner of the other toy, which remained in its original spatial location (“familiar” place). Each mouse was put back into its arena and allowed to explore for 5 ​min. Mouse behavior, measured by exploration time was recorded and scored by Ethovision software. Exploration time was defined as a mouse's nose being within 2 ​cm of either the familiar or novelly located object, but excluding the time the mouse spent on top of the toy. The discrimination index (DI) was calculated for each animal using the equation: ([Novel location exploration time/Total exploration time] – [Familiar location exploration time/Total exploration time]) ​× ​100.

### Elevated plus maze testing

**Fig S1:**
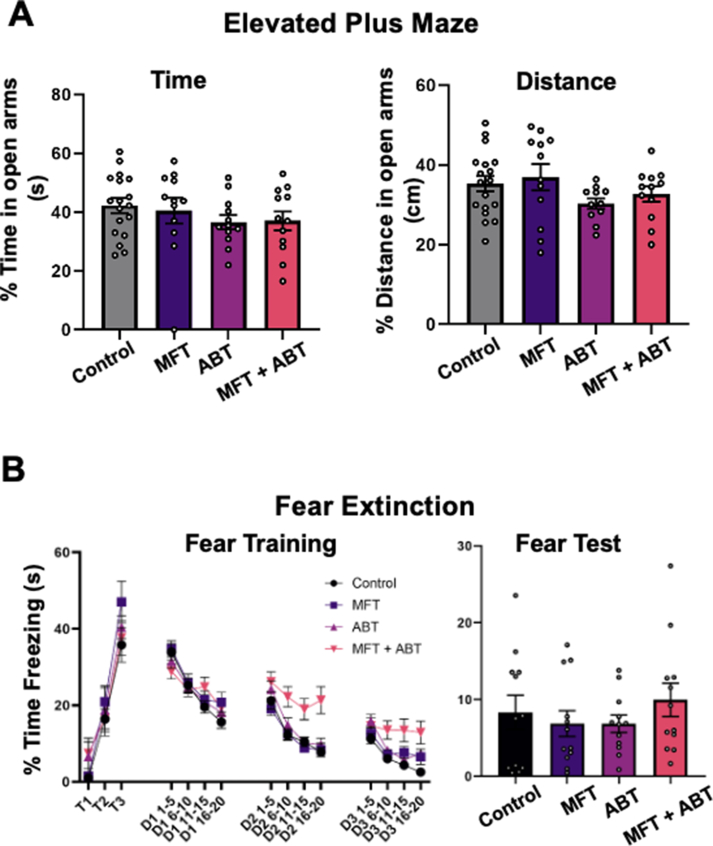
**MFT treatment did not effect behavioral changes all endpoints. (A)** No anxiety-like behaviors were observed 1 month following MFT treatment using the elevated plus maze (EPM) anxiety text or on **(B)** conditioning learning and memory consolidation using the fear extinction test (FE). N = 12–18 mice/group.

Following completion of the PB, OFT, and NPR behavioral testing mice underwent elevated plus maze (EPM) and fear extinction (FE) testing (Suppl. Fig. 1). The EPM is a behavioral test that measures anxiety. The EPM apparatus was comprised of an acrylic surface with four elevated arms (75 ​cm above the floor, 110 ​cm long and 10 ​cm arm width) with two opposing arms enclosed with 42 ​cm high walls (*i.e.,* closed arms) [32]. The maze was subdivided into five different zones: two open arm zones, two closed arm zones and a central zone (10 ​× ​10 ​cm) where the arms intersect. Prior to testing each mouse, the floor and walls of the maze were cleaned with odor elimination disinfectant (Virkon, University Laboratory Animal Resources) and then dried with paper towels. Each mouse was placed in the neutral center zone and allowed to explore for 5 ​min. Testing was recorded with the same camera hardware and analyzed with accompanying tracking software (Noldus, Ethovision XT 17). The percent total time spent in the open arms was compared across experimental groups as a proxy for anxious behavior.

### Fear extinction memory

To determine whether MFT affects fear conditioning learning and fear memory consolidation, we performed fear extinction behavior reliant on hippocampal function [33]. Testing occurred in a behavioral conditioning chamber (17.5 ​× ​17.5 ​× ​18 ​cm, Coulbourn Instruments) with steel shock floors (3.2 ​mm diameter slats, 8 ​mm spacing), and a waste collection tray sprayed with 10 ​% vinegar. For the initial fear conditioning phase (Day 1), mice were allowed to habituate to the chamber for 2 ​min. Three pairings of an auditory conditioned stimulus (16 ​kHz tone, 80 ​dB, lasting 120 ​s; CS) co-terminating with a foot shock unconditioned stimulus (0.6 ​mA, 1 ​s; US) were presented at 2-min intervals. On the following three days of extinction training (Days 2–4), mice were initially habituated to the same context for 2 ​min before being presented with 20 non-US reinforced CS tones (16 ​kHz, 80 ​dB, lasting 120 ​s, at 5 ​s intervals). On a final day of fear testing (Day 5) mice were presented with only three non-US reinforced CS tones (16 ​kHz, 80 ​dB, lasting 120 ​s) at 2-min intervals in the same context. Freezing behavior was recorded with a camera mounted above the chamber and scored by an automated measurement program (FreezeFrame, Coulbourn Instruments). FreezeFrame algorithms calculated a motion index for each frame of the video, with higher values representing greater motion. An observer sets the motion index threshold for each animal individually, which determines what behavior is considered immobility and what is considered motion. The percentage of time each mouse spent freezing was calculated for each day of fear extinction.

### Immunohistochemistry

Immediately after behavior studies were completed, immunohistochemical (IHC) analyses were performed on a subset of the same mice that had been used in the behavior studies. Mice were deeply anesthetized using isoflurane and euthanized via intracardiac perfusion using 1 ​× ​PBS +10 U/ml heparin followed by 4 ​% paraformaldehyde (Sigma-Aldrich) in 100 ​mM phosphate buffered saline. Brains were cryoprotected (10–30 ​% sucrose gradient) and sectioned coronally into 30 ​μm using a cryostat (Leica Microsystems, Germany).

For each endpoint, 2 representative coronal brain sections per animal were selected at approximately 15 section intervals and stored in phosphate buffered saline with sodium azide (PBS, 100 ​mM, pH 7.4, Sigma-Aldrich, St. Louis, MO).

For ChAT (choline acetyltransferase), PSD-95 (post-synaptic density protein 95), and ADK-GFAP (glial fibrillary acidic protein), tissue sections were washed in 3 times 1 ​× ​TBS (pH 7.4) followed by washes in 1 ​× ​TBS-0.1 ​% Triton-X 100. Following blocking, sections were IHC labeled for ChAT-NeuN using Goat anti-ChAT primary antibody (1:500; Millipore) along with Mouse anti-NeuN (1:500, Millipore) in 3 ​% normal donkey serum (NDS) with Tris-A buffer; PSD-95 using mouse anti-PSD-95 primary antibody (1:1000; Fisher Scientific) with 2 ​% BSA in Tris-A buffer, and ADK-GFAP dual immunolabeling using rabbit anti-ADK (1:2000; BETHYL Labs) in combination with mouse anti-GFAP (1:500; Invitrogen) with 3 ​% normal donkey serum (NDS) in Tris-A. Twenty-four hours later the tissues were washed with 1 ​× ​TBS and incubated with secondary antibodies to visualize the target antigens (1:500 Donkey anti-Goat AF 568, 1:500 Donkey anti-Mouse AF 488, 1:1000 Donkey anti Mouse AF 647, 1:400 Donkey anti Rabbit AF 448, and 1:500 Donkey anti Mouse 568, respectively for ChAT, PSD-95, ADK, and GFAP; Invitrogen).

For CD68-IBA1, sections were washed with 1 ​× ​PBS-0.3 ​% Tween 20 and then incubated in 1 ​× ​PBS-3% hydrogen peroxide-1% methanol, followed by a 1 ​× ​PBS wash and blocking in 1 ​× ​PBS-4% BSA-03 ​% Tween-20. Following blocking, hippocampal sections were IHC labeled for CD68 (1:500, rat anti-mouse CD68, Bio-Rad) and IBA-1 (1:500 rabbit anti IBA-1, FUJIFILM Wako) in 1 ​× ​PBS -1 ​% BSA-0.3 ​% Tween-20 buffer. For medial prefrontal cortex (mPFC) specific CD68 staining, primary antibody concentrations were 1:1000 (rat anti mouse CD68) combined with 1:500 (rabbit anti IBA-1). Twenty-four hours later the tissues were washed and stained in secondary antibodies for CD68 and IBA-1 using goat anti-rat AF 647, 1:1000 (Abcam) and goat anti-rabbit AF 488, 1:500 (FisherSci) for CD68 and IBA-1, respectively.

For the A1R and A2aR immunostaining, sections were washed with 1 ​× ​PBS and blocked with 1 ​× ​PBS-10 ​% NDS-0.1 ​% Triton X-100. Following blocking, sections were immunofluorescently labeled for A1R and A2aR using primary antibodies in 1 ​× ​PBS-2% NDS-0.1 ​% TTX (1:200 rabbit anti-A1R, 1:200 rabbit anti-A2aR; Abcam) and visualized using donkey anti-rabbit Alexa Fluor 568 (1:500; Abcam).

For all stains, tissues were then washed and DAPI nuclear counterstained (1 ​μmol/L, FisherSci) and mounted on Superfrost slides (FisherSci) using VectaShield antifade mounting medium (VectaShield).

### Microscopy and 3D algorithm-based volumetric quantification

Immunostained coronal brain sections were scanned at high-resolution (2048p) using a laser-scanning confocal microscope (Nikon AX) equipped with a 20 ​× ​PlanApo lens (0.75 NA, Nikon) or 40 ​× ​oil-immersion Plan Fluor lens (1.3 NA, Nikon) and a NIS-Elements AR interface (v6.02.03, Nikon). Each brain section was imaged using 24 z-stacks 0.5 ​μm thick (442 ​× ​442 ​× ​12 μm) from the hippocampal dentate gyrus and CA1 subfields, and infralimbic medial pre-frontal cortex (mPFC). High-resolution images (16-bit) were processed, and 3-D volume surfaces were created for each antigen of interest using Imaris, an image analysis software (v10.1.0, Oxford Instruments). The 3D algorithm-based surface rendering and quantification of fluorescence intensity for each marker was carried out at 100 ​% rendering quality. Each channel was analyzed separately. 3D surface rendering detects immunostained markers or DAPI nuclear staining, satisfying pre-defined criteria. Dependent on the batch of molecular markers, a set of image processing parameters (*i.e.* baseline subtraction, gamma correction, etc.) was applied to each deconvoluted confocal z-stack volume for all experimental groups including control. The pre-set parameters were kept constant throughout the subsequent analysis of immunoreactivity for each antigen. For ChAT-NeuN, A1R and A2aR, the total surface volume of each marker was obtained and analyzed. For PSD-95, the surface volumes of synaptic puncta were obtained and analyzed. For CD68-IBA1 and ADK-GFAP, co-localization volume between the surfaces of two markers for each stain were determined and analyzed. To maintain uniformity for each antigen analyzed, the volume of each antigen of interest per 442 ​× ​442 ​× ​12 ​μm was normalized to control and data was expressed as a mean percent immunoreactivity relative to untreated controls.

### Statistical analysis

Statistical analyses were carried out using GraphPad Prism (v8). Behavioral data were analyzed by unpaired Student's t tests and one- or two-way analysis of variance (ANOVA) followed by the Bonferroni multiple comparisons test, as appropriate. For all immunohistochemical analyses, one-way ANOVA was used to assess the significance between control and groups of mice receiving MFT, ABT, and MFT ​+ ​ABT. When overall group effects were found to be statistically significant, the Bonferroni's multiple comparisons test was used to reveal differences among experimental groups. For groups with N ​< ​8 the Kruskall-Wallis (non-parametric one-way ANOVA) followed by Dunn's multiple comparisons test was used. Outliers, defined as outside 2 standard deviations of the mean, were removed from statistical analyses. All results are expressed as mean ​± ​SEM. A P value of ≤0.05 was considered statistically significant.

## Results

### Neuromuscular and locomotor effects of MFT exposure

**Fig. 2:**
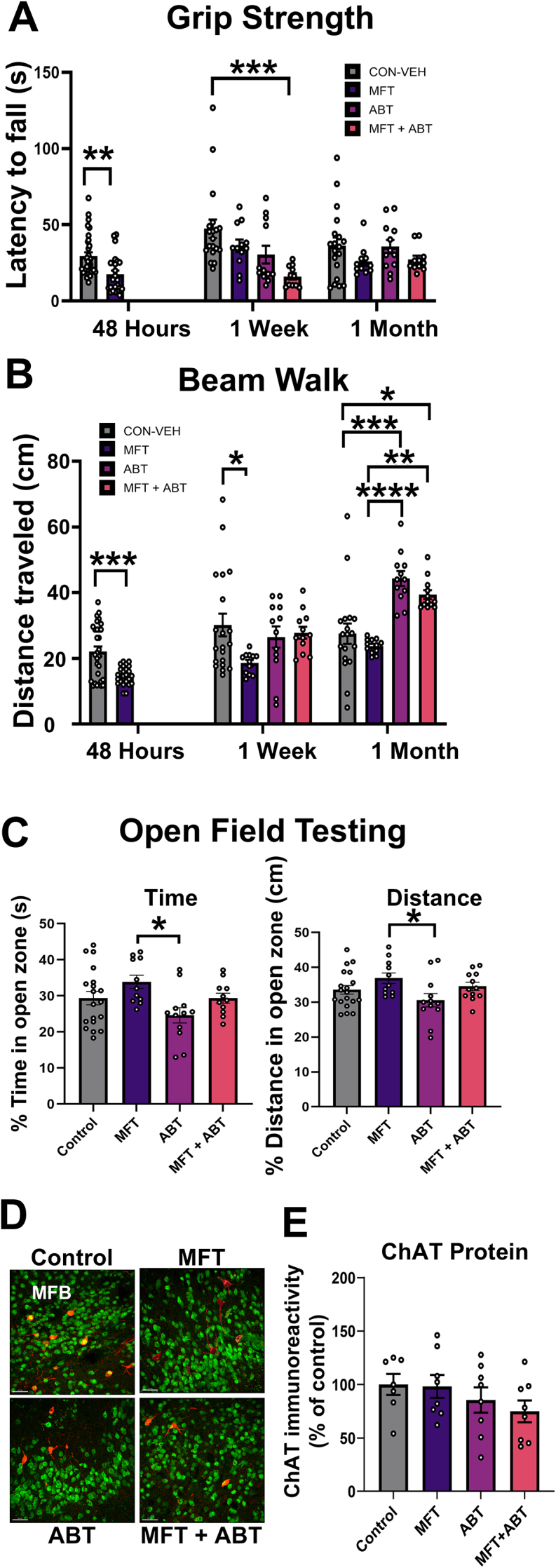
MFT impaired motor function shortly after exposure but did not alter choline acetyltransferase levels one month later. **(A)** Mice exposed to MFT experienced neuromuscular dysfunction exhibiting shorter latency to fall times on the grip strength test 48 ​h post-exposure, but subsequently recovered. **(B)** Similarly, MFT treated mice exhibited locomotor deficits as measured by the beam walk test, covering significantly less distance on the beam than untreated mice at 48 ​h post-exposure that persisted to 1 ​wk post-treatment. Irrespective of MFT treatment, ABT-treated mice walked significantly further than the control and MFT alone mice. **(C)** At 1-month post-treatment, all mice spent relatively similar percents time and distance traveled in an open area as a measure of exploratory behavior. **(D, E)** Representative images and quantitative measures of choline acetyltransferase (ChAT) levels indicate no change as a result of MFT and/or ABT treatment 1 month later suggesting no significant change of acetylcholine levels within the brain. (medial forebrain bundle (MFB); ChAT ​= ​red, NeuN ​= ​green, Scale bar ​= ​40 ​μm). Data are presented as mean ​± ​SEM. N ​= ​23–31 mice/group for the 48 ​h grip strength and beam walk tests and N ​= ​11–20 mice/group at 1 ​wk and 1 ​mo post-treatment for the grip strength and beam walk tests. N ​= ​4 mice/group and 2 sections/mouse for immunohistochemical analysis. ∗P ​≤ ​0.05; ∗∗P ​≤ ​0.01; ∗∗∗P ​≤ ​0.001.

In this study, 56 adult male wild type C57Bl/6J mice were used in a treatment paradigm resulting in N ​= ​20 untreated control mice, 12 ​MFT-treated mice, 12 ABT-treated mice and 12 ​MFT ​+ ​ABT-treated mice. Forty-eight hours after the 24 ​MFT-treated mice received a single dose of MFT (80 ​mg/kg, i.p.) all groups of mice were administered the grip strength and beam walk tests to evaluate forelimb neuromuscular strength and locomotor functions (Fig. 1C) [27,28]. The next day, and for 5 subsequent days, 12 mice (ABT group) and 12 ​MFT-treated mice (MFT ​+ ​ABT group) received daily i.p. injections of ABT (1.6 ​mg/kg). At the end of the ABT treatment, 1-week and 1-month post-MFT treatment, all mice were again administered the grip strength and beam walk tests. For the grip strength test a significant decrease in muscular strength was observed at 48 ​h for the MFT treated mice (Fig. 2A; P ​= ​0.0017), but the mice recovered and performed similarly to control mice and ABT treated mice at 1 week and 1 month post treatment. While the reason is unclear, the MFT ​+ ​ABT mice showed impaired grip strength relative to controls at 1-week post-treatment that was not observed 1 month later (P ​= ​0.0001). The beam walk test revealed that MFT-treated mice exhibited impaired locomotor function relative to control mice 48 ​h and 1 week after MFT treatment (Fig. 2B; P ​= ​0.0003 and P ​= ​0.013, respectively). Interestingly, at 1 month post treatment, the MFT treated mice performed at control levels but that both the ABT and MFT ​+ ​ABT treated groups outperformed both control and MFT treated mice (control vs ABT P ​= ​0.0001, control vs MFT ​+ ​ABT, P ​= ​0.013; MFT vs ABT and MFT ​+ ​ABT, P ​< ​0.0001 and P ​= ​0.0016, respectively).

After the 1-month grip strength and beam walk tests, the OFT was administered. This less challenging measure of locomotor function evaluates the activity of the mice in an open arena, comparing the percentage of time spent in the central 60 ​% of the arena vs hugging the perimeter of the arena. The OFT revealed a decrease in time spent in the central zone and distance traveled for the ABT-treated mice as compared to the MFT-treat mice, that was not reflected in other groups (Fig. 2C; P ​= ​0.015 and P ​= ​0.041, respectively).

To gain insights into the acetyl choline metabolism target of MFT and ABT, we evaluated expression of choline acyl transferase (ChAT) enzyme in the medial forebrain bundle (MFB), which controls locomotor function [34]. ChAT was shown to be unaffected by MFT or ABT treatments relative to controls at delayed post-MFT exposure times, in agreement with the grip strength, beam walk and OFT test results (Fig. 2D and E). Together, these data suggest that any adverse effects of MFT exposure on open arena, spontaneous exploration and problem-solving tasks using NPR and PB are a result of impairments in spatial memory or executive function rather than locomotor function.

### Effects of MFT exposure on cognitive function

**Fig. 3:**
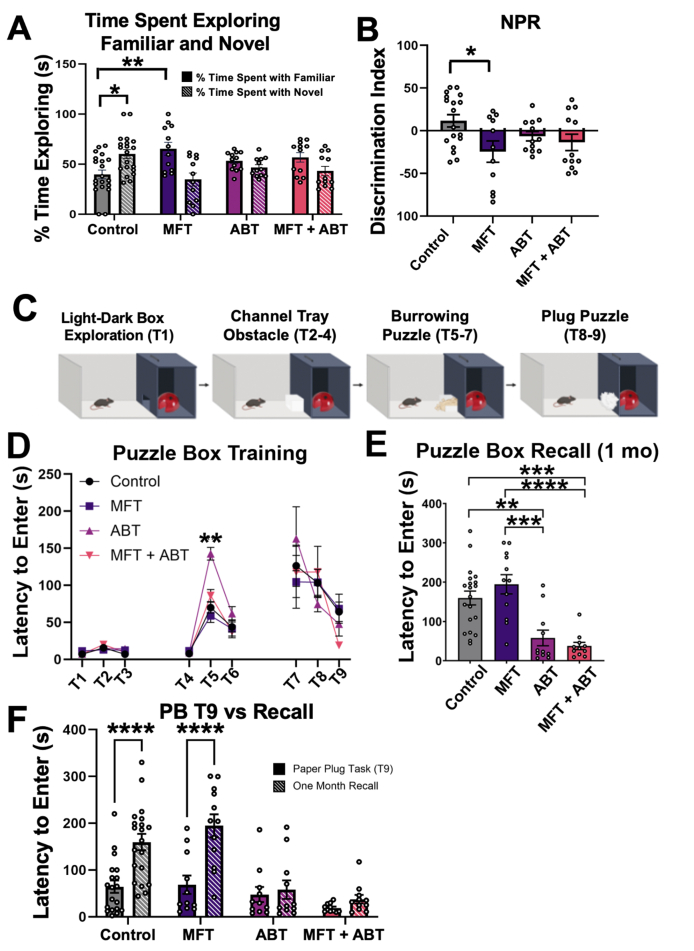
MFT exposure impairs spatial learning and memory. **(A)** Control mice spent the most time exploring the novel location relative to the familiar location, compared to all other groups. **(B)** The discrimination index for novel exploration is calculated as [Novel Time/Total Exploration Time – Familiar Time/Total Exploration Time] ​× ​100. MFT-treated mice displayed a significant negative discrimination index, indicating hippocampal impairments in spatial memory. **(C)** Schematic design of the puzzle box task. After a habituation period (T1), mice had to complete three tasks of increasing difficulty to enter the goal zone: channel (T2-4), bedding (T5-7), and paper ball (T8-9). Within the goal zone was an igloo that served as an incentive for the mice to enter. T(1–3) takes place on Day 1, T(4–6) takes place on Day 2, and T(7–9) takes place on Day 3. **(D)** Out of the three tasks, mice from all groups completed the channel task with similar latencies. Mice given ABT had the longest latency when they encountered the bedding task for the first time, but all cohorts ultimately learned both that task and the paper ball task with similar latencies. **(E)** For the 1-month recall test, ABT ​± ​MFT treated mice demonstrated the lowest latencies for completing the paper ball task, while MFT-treated mice performed similarly to control mice. **(F)** Comparison of T9 and recall test latency showed that both ABT treated groups performed similarly on the memory recall later, while the control and MFT groups were not only impaired at the 1-month recall test, relative to the ABT groups, they performed much worse than they had at T9. Data are presented as mean ​± ​SEM (N ​= ​19–20 control and 11–12 experimental mice/group). P values are derived from two-way ANOVA and Bonferroni's multiple comparisons test. ∗P ​≤ ​0.05; ∗∗P ​≤ ​0.01; ∗∗∗P ​≤ ​0.001; ∗∗∗∗P ​≤ ​0.0001.

During the NPR test phase, a comparison of the percentage time mice explored familiar or novel object placement showed that only the control animals showed a significant appreciation for the novel object placement, over the familiar (Fig. 3A; P ​= ​0.048). The MFT-treated mice showed a decrease in the time spent exploring the novel object placement relative to the familiar, however this did not reach statistical significance. It was also noted that the percent novel object exploration was significantly lower in the MFT-treated animals relative to controls (Fig. 3A; P ​= ​0.0011). When this preference for novel place recognition was calculated as a Discrimination Index (DI) the MFT-treated mice exhibited significantly lower DIs compared to control mice, while the ABT and MFT ​+ ​ABT treated mice were not different from controls (Fig. 3B; P ​= ​0.027). Finally, mice were challenged to a problem-solving task to evaluate executive function using the PB test where mice are given increasingly difficult obstacles to gain access to the safe dark compartment containing an igloo in the goal area separate from the brightly lit portion of the arena (Fig. 3C). Higher latency times taken to reach the goal indicate impairment or lack of interest in solving the presented puzzle. Each day the mice were presented first with a repetition of the previous day's task before being presented with the new more challenging task. During this phase of the PB test mice exhibited similar native problem-solving ability among all groups upon encountering the new challenge for the first time (Fig. 3D; T2, T5, and T8) and achieved similar latency times to enter the goal zone by the third trial each day (Fig. 3D; T3, T6, and T9). A recall test requiring removal of the paper plug to reach the goal zone, a problem identical to that encountered in T8 and T9, was performed 1 month after completion of PB learning, to assess memory retention as compared to the same animal's latency time at T9 (Fig. 3E). The recall test results showed that both control and MFT-treated mice had higher latency to enter times as compared to either of the ABT or MFT ​+ ​ABT treated groups (control vs ABT or MFT ​+ ​ABT P ​= ​0.0022 and P ​= ​0.0002, respectively; MFT vs ABT or MFT ​+ ​ABT P ​= ​0.0002 and P ​< ​0.0001, respectively). Interestingly, the recall test results also indicated that the control and MFT-treated groups both had poorer memory retention when comparing latency to enter at T9 to recall test day latency (P ​< ​0.0001), while both ABT treated groups exhibited similar levels of recall between T9 and recall test day (Fig. 3F). As with the 1-month beam walk test results (Fig. 2B), these PB data suggest that irrespective of MFT treatment, ABT treatment may enhance performance on this test.

Our battery of behavioral testing was concluded using the elevated plus maze (EPM) that determines whether an animal prefers exploring the open or closed arms of the elevated maze under bright lighting as a measure of anxiety-like behavior (Suppl. Fig. 1A). EPM testing was followed by fear extinction (FE) to assess conditioning learning and fear memory consolidation (Suppl. Fig. 1B). No differences were found among any groups for time spent or distance traveled in the open arms of the EPM, nor were any differences among groups observed for fear learning and memory consolidation using FE.

### Molecular effects of MFT exposure

**Fig. 4:**
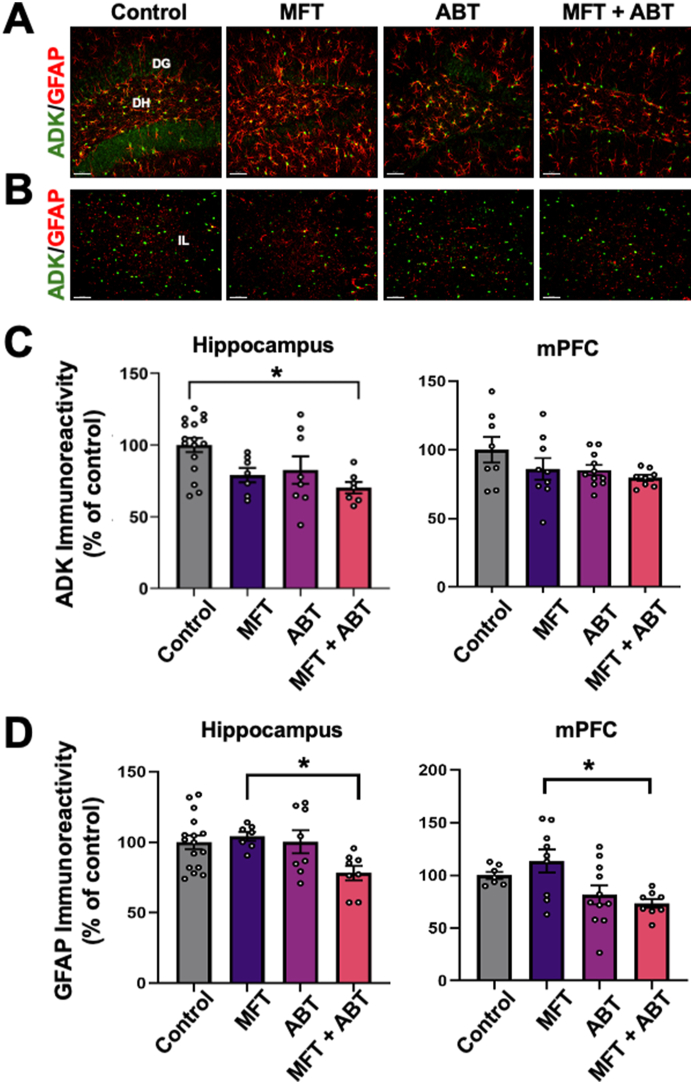
ABT-702 treatment following MFT exposure reduces ADK expression and reduces astrogliosis in the brain 1 month post exposure. Dual-immunofluorescence microscopy images of ADK-GFAP in the **(A)** hippocampus and **(B)** medial prefrontal cortex (mPFC). **(C)** Adenosine kinase (ADK) levels were reduced in the hippocampal region of the brain after MFT ​+ ​ABT treatment. **(D)** Elevated astrogliosis in the MFT-treated brain as measured by GFAP was significantly mitigated by ABT treatment in both regions of the brain. (dentate gyrus (DG), dentate hilus (DH), infralimbic cortex (IL); ADK, green; GFAP, red, Scale bar ​= ​50 ​μm). Data are presented as mean ​± ​SEM (N ​= ​4–8 mice/group). P values are derived from one way ANOVA and Bonferroni's multiple comparisons test. ∗P ​≤ ​0.05.

ADK and GFAP expression were evaluated by IHC as a measure of astrogliosis [18] and intracellular changes as a result of MFT exposure with and without ABT treatment (Fig. 4). While not different from MFT-treated mice, astrocytic ADK expression was reduced in the hippocampus of MFT ​+ ​ABT treated mice relative to control mice (Fig. 4C; P ​= ​0.014). These analyses also revealed that MFT induced astrogliosis in both hippocampal and mPFC regions of the brain as measured by GFAP that was mitigated by ABT treatment (Fig. 4D; P ​= ​0.035 and P ​= ​0.028, respectively).

**Fig. 5:**
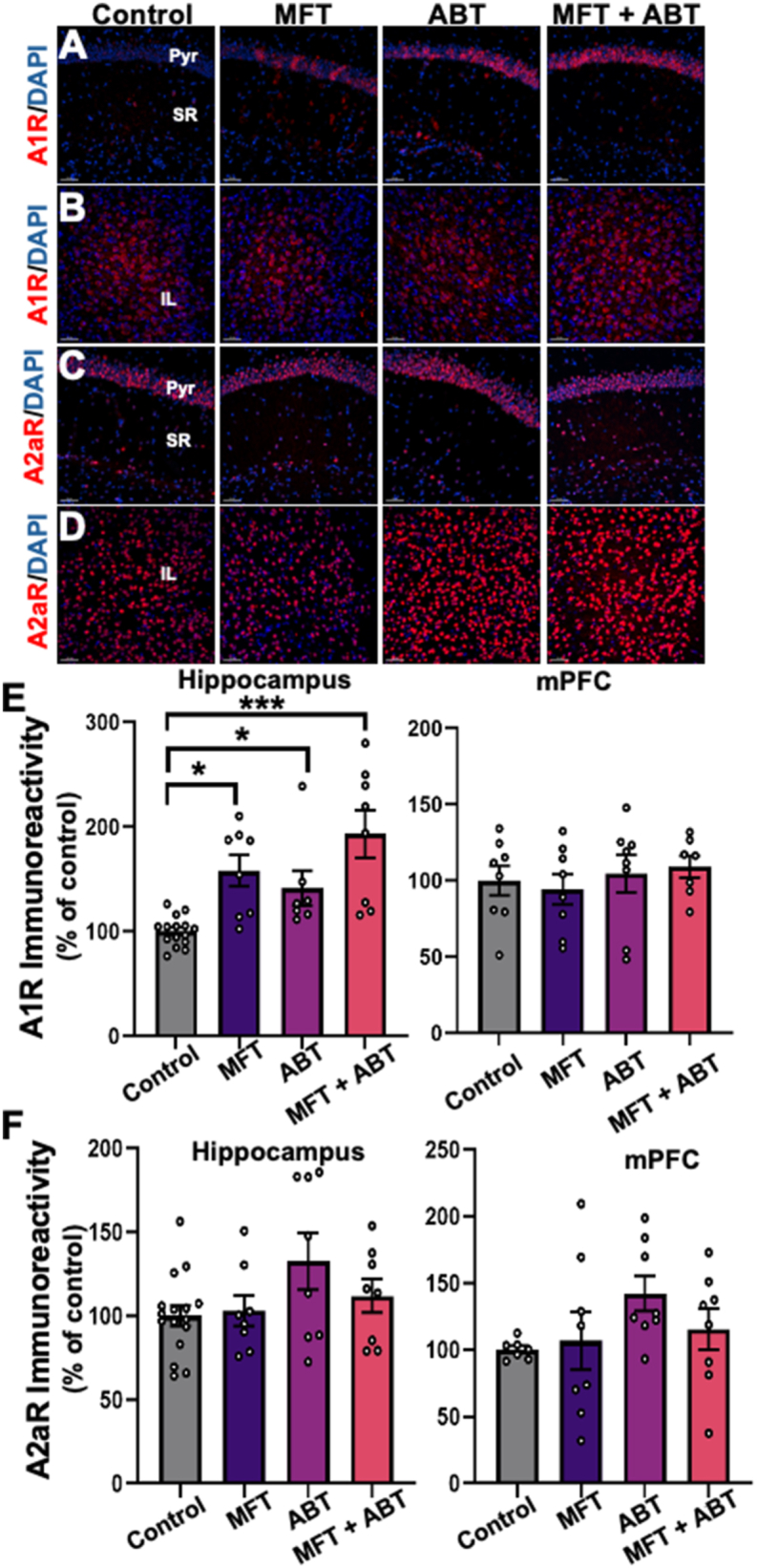
Region specific changes in A1 receptor levels in the brains of mice treated with MFT ​± ​ABT-702 1 month post exposure. **(A)** Representative images indicated that MFT and ABT treatments significantly increased A1R in the hippocampus, but **(B)** not in the mPFC region of the brain. Volumetric quantification of changes in A1 receptor levels (A1R, inhibitory) support these observations **(E). (C, D, F)** No effect of MFT ​± ​ABT treatments were observed in either region of the brain for A2a (excitatory) receptor levels. (dentate gyrus (DG), dentate hilus (DH), infralimbic cortex (IL); A1R and A2aR, red; DAPI, blue, Scale bar ​= ​50 ​μm). Data are presented as mean ​± ​SEM (N ​= ​4–8 mice/group). P values are derived from one way ANOVA and Bonferroni's multiple comparisons test. ∗P ​≤ ​0.05; ∗∗P ​≤ ​0.01.

Astrocytic ADK regulates the levels of synaptic adenosine that exerts its neuromodulatory impact via A1R and A2aR. The A1R propagates inhibitory effects while the A2aR exerts excitatory effects when activated [35,36]. For this reason, we evaluated the levels of these receptors in the hippocampus and mPFC of the MFT-exposed brain (Fig. 5). In the hippocampus, all experimental groups showed significant increases in A1R immunoreactivity relative to controls (Fig. 5 A, E; control vs MFT, ABT and MFT ​+ ​ABT, P ​= ​0.016, P ​= ​0.036, and P ​= ​0.0002, respectively), while no changes among groups were observed in the mPFC region of the brain (Fig. 5 B, E). No changes in A2aR levels were observed among treatment groups in either region of the brain (Fig. 5C, D, F). When evaluated in the context of the decreased levels of ADK in the hippocampus (Fig. 4), particularly the MFT ​+ ​ABT treated group it is unclear whether the increase in receptor protein levels is a compensatory response to the reduced levels of ADK.

**Fig. 6:**
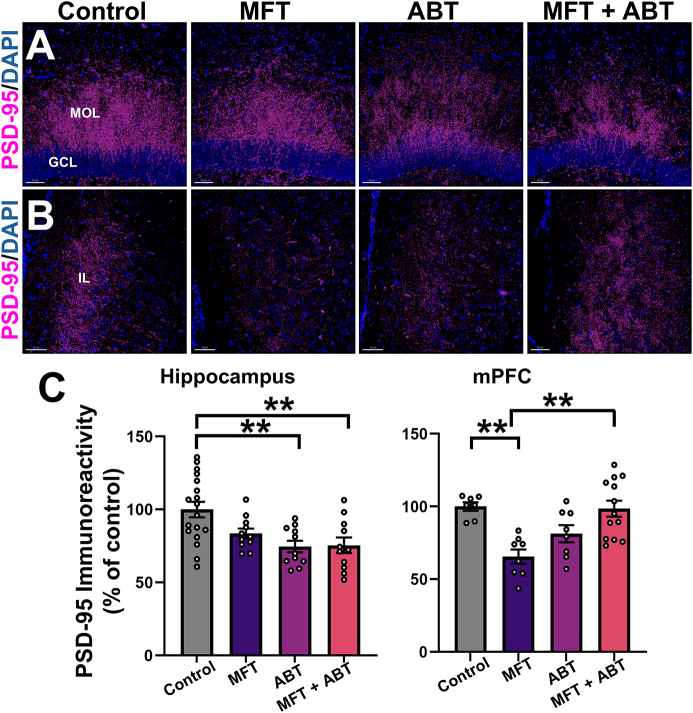
At 1 month post exposure ADK inhibition restored post-synaptic density in the medial prefrontal cortex of MFT-exposed mice. **(A, B)** Representative images of PSD-95 expression in the hippocampus and mPFC, respectively. **(C)** Quantitative assessment of PSD-95. (molecular cell layer (MOL), granuale cell layer (GCL), infralimbic cortex (IL); PSD-95, magenta; DAPI, blue; Scale bar ​= ​50 ​μm). Data are presented as mean ​± ​SEM (N ​= ​4–8 mice/group). P values are derived from one-way ANOVA and Bonferroni's multiple comparisons test. ∗P ​≤ ​0.05; ∗∗P ​≤ ​0.01.

Postsynaptic protein-95 (PSD-95) is a scaffolding protein that regulates the trafficking and localization of glutamate receptors within the postsynaptic membrane [37]. Changes in PSD-95 expression levels are indicative of changes in synaptic development and plasticity. While the MFT-treated mice exhibited a decrease in PSD-95 immunoreactivity within the molecular layer of the hippocampus compared to the control group, it did not reach statistical significance. However, both the ABT and MFT ​+ ​ABT treated mice both showed significantly reduced PSD-95 immunoreactivity (Fig. 6 A, C; P ​= ​0.003 and P ​= ​0.0044, respectively). We also analyzed the immunoreactivity (volume) of PSD-95 in the mPFC, a region predominantly associated with cognitive reasoning as well as other higher-order brain functions [38]. MFT significantly reduced PSD-95 immunoreactivity compared to the control group and the ABT treatment of MFT exposed mice restored levels of PSD-95 to that of controls (Fig. 6 B, C; P ​= ​0.0048 and P ​= ​0.0048, respectively).

**Fig. 7:**
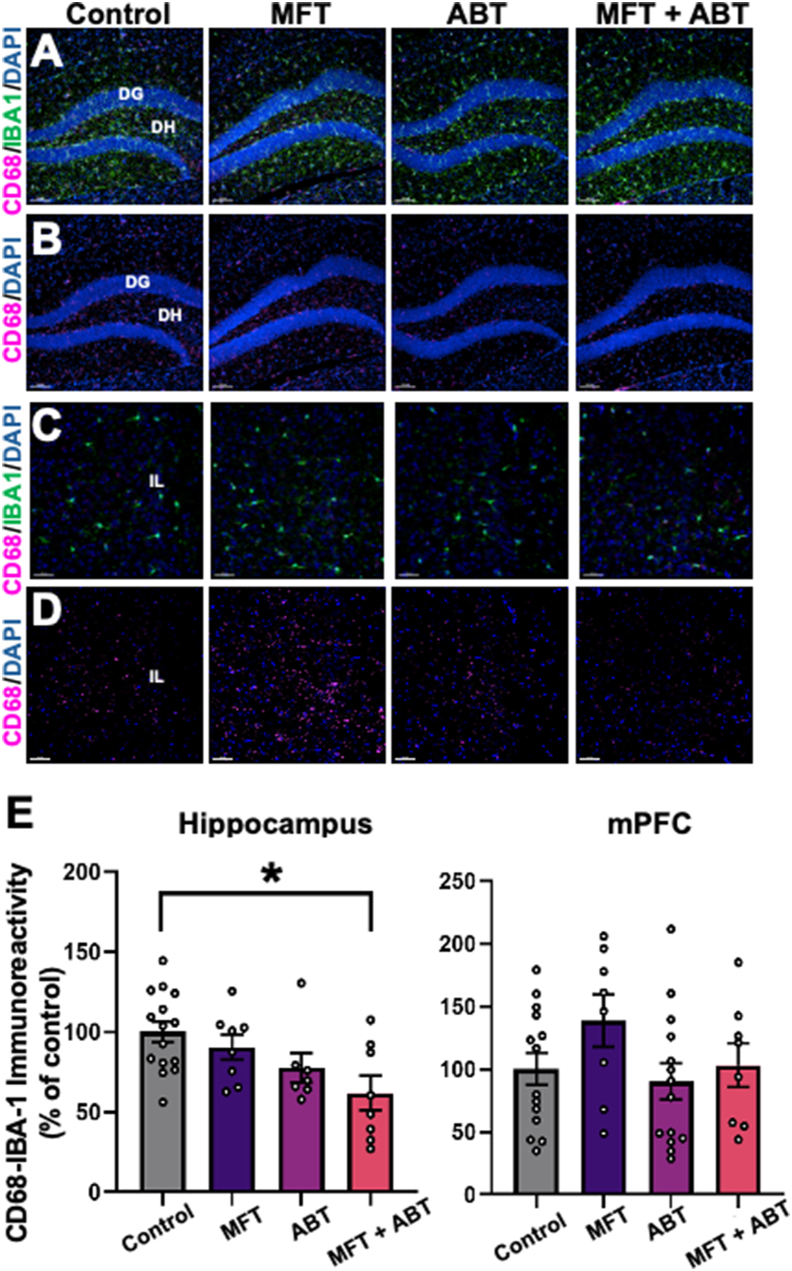
MFT ​± ​ABT treatment induced region-specific changes in neuroinflammation in the exposed brain. **(A–D)** Representative images for CD68/IBA-1 colocalization and CD68 alone in the hippocampus (**A, B**) and the mPFC (**C, D**). Quantitative 3D volumetric analysis of the colocalization volume of microglial marker IBA-1 and lysosomal protein CD68 demonstrated that MFT-treatment resulted in a trend for elevated microglial activation in the mPFC that did not reach statistical significance. CD68, magenta; IBA-1, green; DAPI, blue, Scale bar ​= ​100 ​μm for hippocampus and 50 ​μm for mPFC. Data are presented as mean ​± ​SEM (N ​= ​4–8 mice/group). P values are derived from one-way ANOVA and Bonferroni's multiple comparisons test. ∗P ​≤ ​0.05.

Given observations in the literature that OP exposures could elicit neuroinflammation [1] led to the evaluation of CD68^+^ and IBA-1^+^ activated microglia in the current study [39] (Fig. 7). MFT treatment did not increase inflammation in the hippocampus (Fig. 7 A, B, E). However, an increase in CD68/IBA-1^+^ dual-labeled cells was observed in the mPFC of the MFT-treated mice, but it did not reach statistical significance (Fig. 7C, D, E). Interestingly, ABT treatment of the MFT-exposed mice resulted in reduced microglial activation relative to controls in both regions of the brain (P = 0.029). These data suggest that if MFT induces any inflammatory response, it occurs and resolves prior to our delayed post-exposure time.

## Discussion

In this study, we tested the hypothesis that low-dose OP exposures might lead to neurotoxic consequences for the CNS that would manifest as cognitive dysfunction, including anxiety, depression, and learning and memory deficits [6]. Because the brain can be a late responding tissue to insult, the adverse effects of exposure might not manifest until delayed times post-exposure and may persist without resolution. Our data support the hypothesis that 10.13039/100017269OP toxicity might pose a long-term significant threat for warfighters, first responders, clean-up workers and civilians alike in accident zones as well as regions of chemical warfare or terrorist attacks. In this scenario, the development of effective countermeasures is critical.

The results of our study using a single acute low dose exposure to MFT demonstrated transient neuromuscular and locomotor impairments that resolved by one month post exposure. This outcome suggests that MFT does not have any long-term adverse effect on neuromuscular strength or locomotor function 1 month after exposure, but that ABT treatment could have some delayed beneficial influence on fine locomotor function that does not similarly influence neuromuscular strength. However, decrements in spatial memory were observed at this 1-month time point. These data suggest that these spatial memory deficits may be directly related to MFT exposure, given the lack of change in neuromuscular strength, locomotion, and open field exploration, as well as ChAT protein levels in the MFT-exposed groups, which play a key role in locomotor function [34].

Although, no significant changes in ADK expression were observed as a result of MFT exposure in the hippocampus or the mPFC, an increase in GFAP was found in the mPFC, suggesting some persistent, region-specific astrogliosis [18]. Given that adenosine modulates synaptic transmission and neuronal activity via A1 and A2 receptors [40,41], it is interesting to note that we also observe significant increases in protein levels in the hippocampus of mice treated with MFT ​± ​ABT relative to controls. While these data do not provide information regarding receptor function, any change in receptor expression may alter excitatory tone at synapses and disrupt homeostasis [11,15]. Similar observations can be made regarding the post-synaptic PDS-95 protein levels that were reduced as a result of MFT exposure, specifically in the mPFC region of the brain, as were trends for increased MFT-related neuroinflammation [1]. While this low acute dose of MFT (80 ​mg/kg) had significant adverse effects on neuromuscular strength and locomotor function 48 ​h post-exposure, it is unclear if other neurobiochemical endpoints were affected at that time. Irrespective, many changes had resolved by one-month post-exposure.

ABT-702 has been shown numerous beneficial effects including protection against diabetic retinopathy [21] or myocardial ischemia [42], provide analgesic and anti-inflammatory efficacy in rat studies of arthritis, thermal hyperalgesia, and paw edema (22; [41,43]. ABT has also been shown to leverage its anti-inflammatory effects in protection of the kidney against streptozotocin-induced diabetes [44]. However, the compelling outcome of this study was the impact of ABT on the brain. We observed that ABT treatment not only improved locomotor function on the beam walk test one month later, but it also improved executive function as measured by the long-term memory recall test using the puzzle box test. Irrespective of MFT treatment, ABT also had at least subtle impacts on ADK levels and astrogliosis, A1R expression, microglial activation and PSD-95 protein levels. These data suggest that ABT may impart anti-inflammatory properties at early times post treatment that might be beneficial, an observation that has been found in other studies of ABT-702 [40,41,43,45].

Overall, this study suggests that MFT-induced impairments observed 48 ​h after exposure are likely to impact the ability of exposed individuals in critical crisis situations. Furthermore, future studies should investigate frontal brain–dependent neurocognitive impairments induced by MFT OP exposure under chronic, high-dose conditions and at additional time points.

## Author contributions

MMA and JEB conceptualized and designed the study; KN, DS, NG, and MGJ developed methodology; KN NG, and MGJ acquired the data; KN, MMA, and JEB analyzed and interpreted the data; KN, MMA and JEB wrote, reviewed and/or revised the manuscript; MMA and JEB provided administrative, technical, or material support; MMA and JEB supervised the study.

## Declaration of competing interest

The authors declare that they have no known competing financial interests or personal relationships that could have appeared to influence the work reported in this paper.
